# Rock Outcrops Redistribute Organic Carbon and Nutrients to Nearby Soil Patches in Three Karst Ecosystems in SW China

**DOI:** 10.1371/journal.pone.0160773

**Published:** 2016-08-10

**Authors:** Dianjie Wang, Youxin Shen, Yuhui Li, Jin Huang

**Affiliations:** 1 Key Laboratory of Tropical Forest Ecology, Xishuangbanna Tropical Botanical Garden, Chinese Academy of Sciences, Menglun, People’s Republic of China; 2 Yunnan Normal University, Kunming, People’s Republic of China; 3 Stone Forest Scenic Area Administration, Shilin, People’s Republic of China; Old Dominion University, UNITED STATES

## Abstract

Emergent rock outcrops are common in terrestrial ecosystems. However, little research has been conducted regarding their surface function in redistributing organic carbon and nutrient fluxes to soils nearby. Water that fell on and ran off 10 individual rock outcrops was collected in three 100 × 100 m plots within a rock desertification ecosystem, an anthropogenic forest ecosystem, and a secondary forest ecosystem between June 2013 and June 2014 in Shilin, SW China. The concentrations of total organic carbon (TOC), total nitrogen (N), total phosphorus (P), and potassium (K) in the water samples were determined during three seasons, and the total amounts received by and flowing out from the outcrops were calculated. In all three ecosystems, TOC and N, P, and K were found throughout the year in both the water received by and delivered to nearby soil patches. Their concentrations and amounts were generally greater in forested ecosystems than in the rock desertification ecosystem. When rock outcrops constituted a high percentage (≥ 30%) of the ground surface, the annual export of rock outcrop runoff contributed a large amount of organic carbon and N, P, and K nutrients to soil patches nearby by comparison to the amount soil patches received via atmospheric deposition. These contributions may increase the spatial heterogeneity of soil fertility within patches, as rock outcrops of different sizes, morphologies, and emergence ratios may surround each soil patch.

## Introduction

Karst landscapes constitute approximately 12–15% of the global terrestrial surface [1–3] and thus represent an important global ecosystem. Rock outcrops are common features of the karst ecosystem [3–5]. A recent report indicated that a 12×10^4^ km^2^ area of karst land was under rock desertification (bedrock exposure ratio ≥30% of the land surface, Technology Regulations of Vegetation Restoration in Karst Desertification Zone, LY/T 1840–2009) which accounts for 26.5% of the total karst terrain in China (Gazette of Rock Desertification in China, 2012). Thus, it has been suggested that large areas of karst lands globally may have rock outcrop proportions greater than 30%. However, little attention has been directed to the ecological function of these outcrops.

These ecological functions of rock outcrops in karst areas merit detailed study. The role of organic carbon and nutrients from rock outcrops in maintaining soil fertility, ecosystem productivity, and biodiversity of soils nearby has been noted in cobbles in deserts [6,7], inselbergs in savanna ecosystems [8], and rocks in pristine glacial forefield sites [9]. Elbert et al. [10,11] also highlighted the contribution of cryptogams (including epiliths) to global C and N cycles. However, little attention has been paid to the ecological functions of rock outcrops in karst areas, which tend to be viewed as negative features that add little productivity, but occupy a great deal of space.

**Fig 1:**
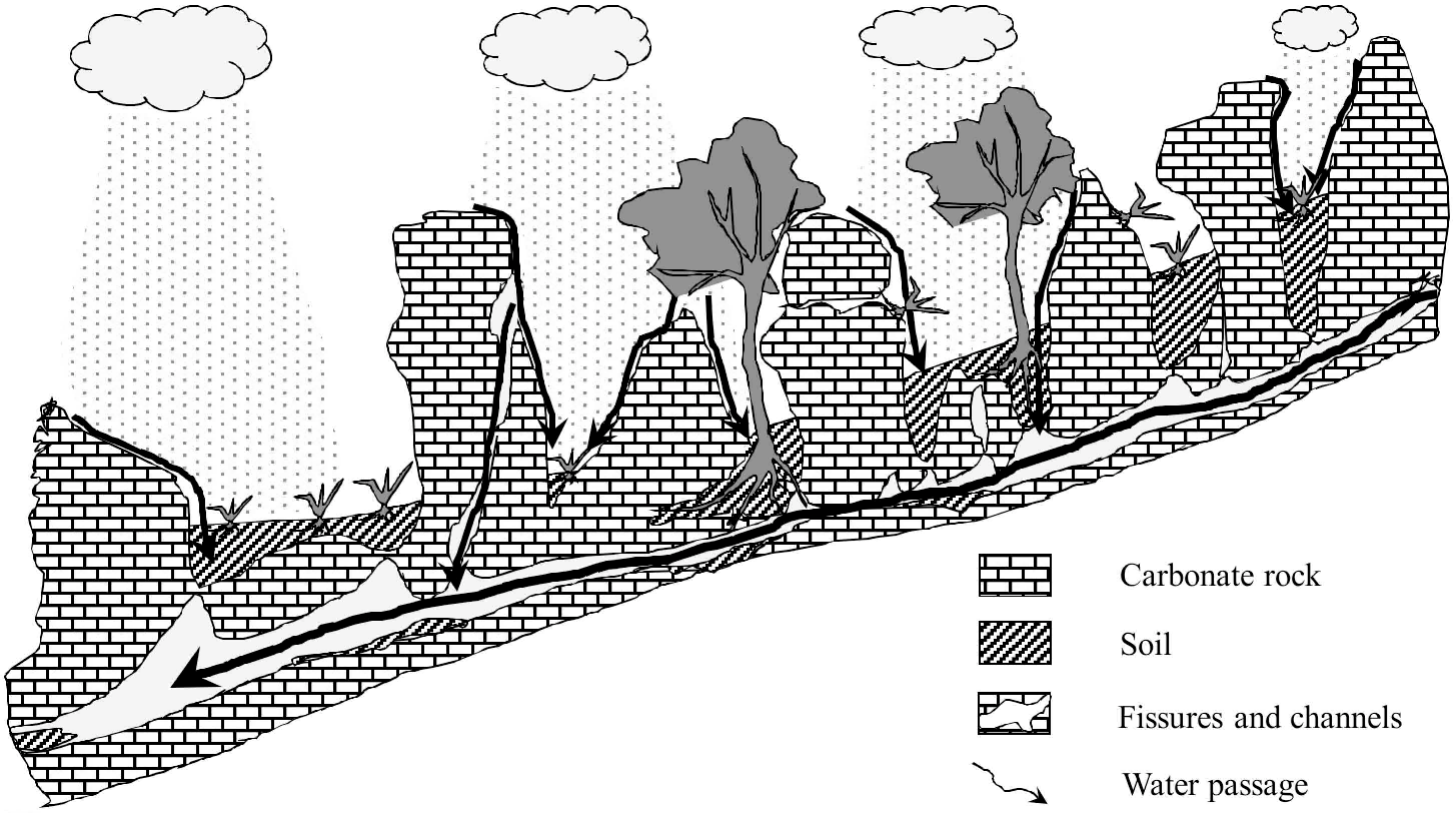
Illustration of the distribution of rock outcrops and soil patches in karst area. Soil patches can support the growth of vascular plants, while various outcrops can receive and redistribute atmospheric water and nutrients to soil patches.

Rock outcrops and epilithic organisms function in concert to fix atmospheric carbon (C) and receive nitrogen (N) depositions [12]. The estimated biomass of chlorolichen growing on limestone on the karst plateau of Trieste, Italy is 30–594 g m^−2^ of ground surface area [13]. Annual net C uptake fluxes of biological crusts on rocks range from 0.6–21.7 g m^−2^ a^−1^ globally [11]. Wang et al. [14] investigated the epilithic organic matter and nutrient contents of three different karst ecosystems in Shilin, China, and found 1.63–32.91 g m^−2^ of organic matter, 0.097–1.59 g m^−2^ of nitrogen (N), 0.006–0.15 g m^−2^ of phosphorus (P), and 0.014–0.22 g m^−2^ of potassium (K) accumulated on rock surfaces. The organic carbon and nutrients are transferred over time via water runoff onto nearby soil patches, thus creating a specific rock outcrop-soil patch system (Fig 1). Soil organic carbon is associated strongly with the soil’s biological and chemical properties [15,16], and the nutrients supplied by soils govern the productivity of terrestrial ecosystems [17,18]. Depending on such factors as the purity of the rock base, local rainfall, and thermal conditions, the land surface may consist of various morphologies of rocky outcrops and rock-soil patterns [2,19,20], and thus may have different patterns of transfer. In regions with extensive rock outcrops, soil–plant systems appear to be influenced by the redistribution of these resources from outcrops.

Our study focused on the chemistry of rock surface runoff onto soil patches nearby. Water received by rock outcrops and the subsequent runoff to soils near rock outcrops were collected and analyzed to measure the concentrations of total organic carbon (TOC), N, P, and K to assess the ecological significance of karst rock outcrops. Our objectives were to: (1) quantify the organic carbon and nutrients exported from rock outcrops in different ecosystems and during different seasons throughout the year; (2) ascertain factors relevant to the export of organic carbon and nutrients, and (3) evaluate the ecological significance of the exports.

## Materials and Methods

### Study area and sites

This study was conducted from June 2013 to June 2014 in Shilin County (24°30′-25°03′N, 103°10′-103°40′), Yunnan Province, SW China, which is characterized by the mid-Permian burial of terrain by continental basalts, exhumation, re-karstification, reburial by Eocene continental deposits, and late Tertiary and Quaternary re-exhumation and re-karstification. Pinnacles ranging from 1–35 m in height and 1–20 m in diameter are common in this area. The rugged tor-and-pediment topography is the most celebrated pinnacle karst in the world [19,21], and more than 400 km^2^ of land is covered with various karst landforms [22]. The central portion is famous for different pinnacle landscapes and became part of the Global Geopark established in 2004. Elevations range from 1700–1900 m, and the region experiences a subtropical monsoonal climate. The mean annual precipitation and temperature are 948 mm and 15.8°C, respectively [22]. Cyanobacteria typically cover exposed rocks, while those under vegetation also develop bryophytes and vascular epiphytes [23–25]. Semi-humid, evergreen, broad-leaved forest with deciduous components, and *Pinus yunnanensis* forest are the major zonal vegetation types [22,26]. Intact primary forest is rare in this region because of human activity. We obtained all necessary permits for the field experiment from the Stone Forest Scenic Area Administration, Shilin County.

Outside the central tourism area, three of the most common karst ecosystems that are representative of SW China [20] and have similar rock outcrop emergence ratios were selected for the study; pinnacles at these specific sites are less than 3 m high.

1. Rock desertification ecosystem (RDE): Local farmers cut down the trees and exposed rock outcrops. Low shrubs and grasses, including *Spiraea salicifolia*, *Heteropogon contortus*, *Themeda triandra*, *Bidens pilosa*, and *Sophora viciifolia*, dominate the plant community. The elevation of the site is 1775 m. Cyanobacteria form discontinuous films on rock surfaces.
2. Anthropogenic forest ecosystem (AFE): Prior vegetation was removed and replaced with *Pinus yunnensis*, *Photinia × fraseri*, *Pyracantha fortuneana*, and *Koelreuteria paniculata*, most of which surpassed the exposed rocks in height and provide partial shade (Table 1). The site elevation is 1789 m, and is located 2150 m from the RDE. Cyanobacteria still dominate the rock surface, but are darker in color than are those in the RDE [23,24].
3. Secondary forest ecosystem (SFE): The dominant tree species are *Cyclobalanopsis glaucoides*, *Pistacia weinmannifolia*, *Neolitsea homilantha*, and *Olea yunnanensis*, mixed with several deciduous components, such as *Pistacia chinensis*, *Albizia mollis*, and *Carpinus mobeigiana*. The ecosystem consists of a karst, semi-humid, evergreen broad-leaved forest that regenerated naturally after being disturbed in the 70s [26]. The forest is dense, has a clear vertical stratification, and outcrops are covered by the tree canopy (Table 1). The site’s elevation is 1918 m, and it is located approximately 21.5 km from the AFE. Cyanobacteria, lichens, bryophytes, and vascular species dominate on rock outcrops in this ecosystem [25].

**Table 1:** General features of the sampled outcrops and the tree canopy coverage.

Features	RDE	AFE	SFE
Height (cm)	123.05±7.03	123.3±10.04	164.7±15.50
Slope (°)	66.44±3.1	73.9±3.63	73.65±2.53
Canopy coverage above rock on 10^th^ August (%)	0.00±0.00 c	23.75±11.68 b	75.00±12.03 a
Canopy coverage above rock on 17^th^ December (%)	0.00±0.00 c	15.75±9.95 b	54.11±13.45 a
Orientation	NE(4) SE(3) SW(2) NW(1)	E(2) NW(2) SE(1) SW(1) NE(1) S(1) W(1) N(1)	NW(2) SE(2) NE(2) SW(1) N(1) E(2)
Organic carbon (g m^-2^ rock surface area)	4.44±0.43 c	28.07±5.33 b	53.48±11.44 a
Total nitrogen (g m^-2^ rock surface area)	0.45±0.04 c	2.02±0.32 b	4.44±0.94 a
Total phosphorus (g m^-2^ rock surface area)	0.029±0.0027 c	0.12±0.021 b	0.4±0.074 a
Total potassium (g m^-2^ rock surface area)	0.07±0.0062 b	0.59±0.104 a	0.85±0.167 a

Different lowercase letters indicate significant differences between two systems (*P* <0.05). Numbers in brackets represent the number of sampling rock surfaces in a specific orientation.

### Selection of rock outcrops and determination of their general features

All rock outcrops were identified within the 100 × 100 m plot that was used in the investigation of local vegetation [25,26], and those within the plot with a sampled surface area of approximately 1 m^2^ were numbered; thereafter, ten numbered rocks were selected randomly in each ecosystem.

Rock height, slope (JZC-B2 portable gradient meter), and orientation (compass) were measured for each of the rocks (Table 1). Because the rock surface is uneven, the mean of six values, three on the top and three on the bottom, was used as the slope for each outcrop sampled. The canopy coverage above each rock sampled was inspected visually on 10 August and 17 December 2013. No significant differences were found between the different ecosystems with respect to rock height and slope (Table 1).

### Water received and delivered from the rock outcrops to soil patches nearby

**Fig 2:**
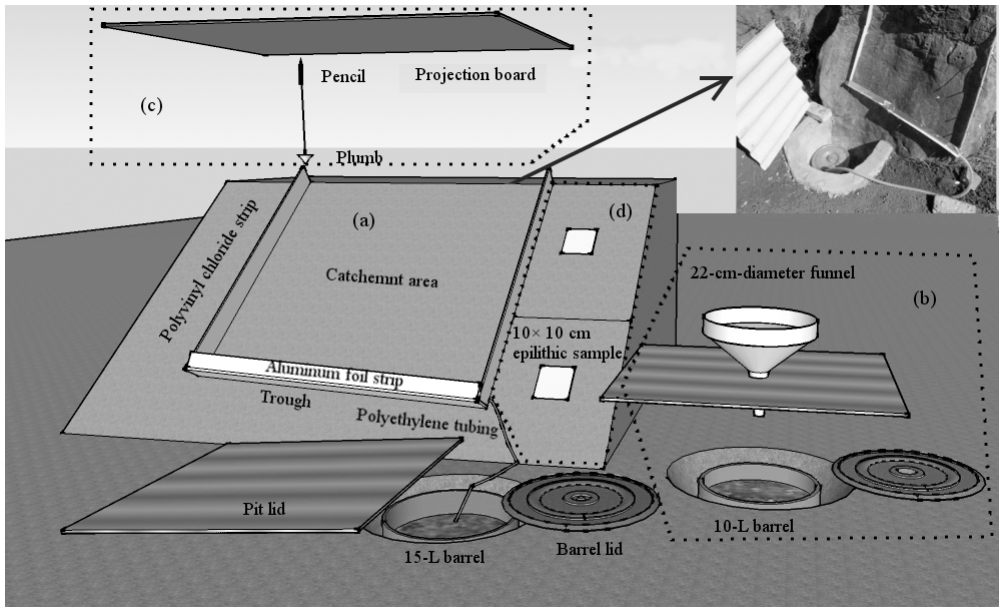
(a) Collection systems for rock runoff water; (b) collection systems for input water falling on rock surface; (c) determination of rock projected area, and (d) sampling of epiliths.

We used a portable cutting machine to create three grooves 0.5–1 cm in depth on the surface of each rock. Two of these ran parallel from the top towards the ground, ending 30–40 cm above the soil. The third groove crossed the lower ends of the first two at an angle of 20–45° to facilitate water drainage. Polyvinyl chloride strips 6.5 cm in width were inserted into the grooves and held in place by a silicon sealant. Aluminum foil strips 4 cm in width were bent into an “L” shape, and were affixed to the bottom edge of the polyvinyl chloride strips to form a trough. The lower end of the trough was attached to a 15-L barrel fitted with polyethylene tubing. The barrel was placed in a pit that was slightly shallower than the depth of the barrel to prevent the collection of runoff from the ground surface (Fig 2a). Water collected by this system was regarded as export that would be redistributed to nearby soil. All the intersections of the system were sealed and tested to ensure that they were impervious. In order to measure the input falling onto the rock surface, a separate 22-cm-diameter funnel was placed above a 10-L barrel, also connected with polyethylene tubing (Fig 2b). These barrels were placed in pits 1–1.5 m from the sampled rock surface, to avoid contamination by water splashing off the rock. The barrels and pits were covered, except for a circular opening to receive the tubing. Polypropylene mesh was used to cover the openings of the trough and the funnel to prevent them from becoming blocked by leaf litter.

The volume of water in the barrels was measured after each heavy rain during the rainy season, and once a month during the dry season. Samples were collected on 30 June 2013, 1 September 2013, and 18 February 2014, and we measured the TOC and nutrient concentrations of the water input and runoff to represent the concentrations from May to July, August to October, and November to April, respectively (Table 2). These three sampling times were scheduled by monitoring local weather forecasts; they did not take place at the exact midpoint of each season. One sample for each seasonal period may not represent the concentration of all rain events well; however, a rain schedule that can be caught and that can produce enough runoff water to be sampled was not easy to follow. Prior to sampling, troughs and barrels were cleaned with deionized water to eliminate any living organisms and inorganic remains. Funnels were not washed, as no attempt was made to differentiate between dry and wet deposition. Water sampled from each barrel was placed in a 550-ml polyethylene sampling bottle (washed with deionized water, and then with water from the barrel to eliminate any dilution effect). To mitigate any effect of bacteria, we packed the 60 samples (30 inputs and 30 exports) on ice in insulated boxes and sent them to the Biogeochemistry Laboratory of Xishuangbanna Botanical Garden, Chinese Academy of Sciences, to perform the chemical tests as quickly as possible. TOC was determined with a TOC analyzer (Vario TOC cube, Elementargroup, Hanau, Germany). Total N was determined via the alkaline potassium persulfate digestion UV spectrophotometric method, and total P was determined using the ammonium molybdate spectrophotometric method, both of which relied on a UV-Visible Spectrophotometer (UV2450, Shimadzu Corporation, Tokyo, Japan). K ion concentrations were determined by means of inductively coupled plasma atomic emission spectroscopy (iCAP6300, Thermo Electron Corporation, Waltham, USA).

**Table 2:** Summary of collection time, volume, number, and chemical analyses for samples.

Date	Volume of single sample (ml)	Number of samples	Type of chemical analyses for each sample
30^th^ Jun	550	60 (Input 10, export 10 for each ecosystem)	Concentration of TOC, Total N, Total P, K ion
1^st^ Sep	550	60 (Input 10, export 10 for each ecosystem)	Concentration of TOC, Total N, Total P, K ion
18^th^ Feb	550	60 (Input 10, export 10 for each ecosystem)	Concentration of TOC, Total N, Total P, K ion

Seasonal and annual inputs of TOC and nutrients, and their exports from each rock surface were calculated by multiplying concentrations by seasonal/annual water quantities (water data can be referred to [27]).

### Areas of rock surfaces sampled

We measured the surface area of the collection system and its projected area (catchment area) to calculate the annual quantity of input and export from outcrops, as well as the epilithic pool of organic carbon and nutrients. A piece of 1.5 × 1.5 m paper was placed on the surface of the sampled area, and its boundary was marked on the paper to outline the surface area; it should be noted that this method might be slightly inaccurate due to the uneven rock surfaces. A 2 × 2 m poster board, to which we attached one piece of white paper of the same size, was prepared as a projection board. A 20 × 20 cm stainless steel plate mounted on top of a 1.7-m-high stainless steel pole supported the projection board. The projection board was placed in a horizontal orientation. A plumb, with the end of the string tied to the bottom of a pencil, was used to trace the sampling boundary. Following the movement of the plumb, the pencil drew an enclosed line on the paper attached to the projection board to represent the projected area of the sampled rock (Fig 2c).

The two papers were numbered and brought to the laboratory to determine the acreage of the marked areas. A semi-transparent paper with a 0.2 cm × 0.2 cm printed grid was placed on the enclosed area, and the acreage was calculated by counting the number of squares within the area. We estimated the values for squares that were enclosed in part.

### Collection of epiliths and determination of their organic carbon and nutrients content

We collected two 10 × 10 cm epilithic samples adjacent to either the left or right strip of the runoff water collection system, one near the upper side of the strip and another near the lower side (Fig 2d). Epiliths did not cover the rock surface evenly, and scraping proved to be an arduous task. Samples from the upper and the lower side were dried and weighed, and mixed to represent the epiliths of the rock. Ten samples from each ecosystem were amalgamated into three and subjected to chemical analysis. TOC was determined by the oil-bath K_2_Cr_2_O_7_ titration method, and total N was determined via the Kjeldahl nitrogen determination method after digestion in concentrated sulfuric acid, using the BUCHI AutoKjeldahl Unit (K370, Switzerland). The K and P content were determined with an ICP-AES after digestion with HClO_4_-HF (iCAP6300, U.S.A.).

The TOC and N, P, and K per m^2^ of surface area were calculated for each rock sampled (Table 1). Based on the ratio of surface to catchment area for each rock, the amount of organic carbon and N, P, and K per unit of projected area (the epilithic nutrient pool) could be calculated.

### Statistical analyses

An ANOVA followed by least significant difference (LSD) multiple comparisons were performed to test for differences in the concentration and quantity of organic carbon and nutrients between seasons and among ecosystems. A paired t-test was conducted to test the differences in concentration and quantity between the input and runoff. Data transformation and the Kruskal-Wallis rank sum and Wilcoxon signed-rank tests were used if prerequisites of normality and homogeneity of variances were not met.

Partial correlation analyses (PCAs) were performed to determine the degree to which various factors affected the organic carbon and nutrient concentrations of the runoff export for each season. Prior to the PCAs, the TOC and nutrient concentrations of exported samples, together with other factors, were transformed to achieve normality.

The ratio between the input and export flux, and the epilithic pool of organic carbon and nutrients of the rock outcrops were used to trace the movement of organic matter and nutrients between the rock surface and input water. Soil patches receive TOC and N, P, and K both from precipitation (soil area × amount of precipitation per m^2^) and from rock runoff (projected sampling area of rock outcrops × amount of runoff per m^2^) under various rock outcrop-soil patch area combinations. The ratio of runoff to precipitation input flux of organic carbon and nutrients to soil was calculated at rock emergence ratios of 30% and 70% to evaluate the significance of TOC, N, P, and K runoff to nearby soil patches. These percentages were selected because 30% is the lower criterion of rock desertification and 70% is the criterion that determines the highest level of rock desertification indicated by the National Forest Bureau of China (Technology Regulations of Vegetation Restoration in Karst Desertification Zone, LY/T 1840–2009).

## Results

### Concentration of organic matter and nutrients in sampled water

**Fig 3:**
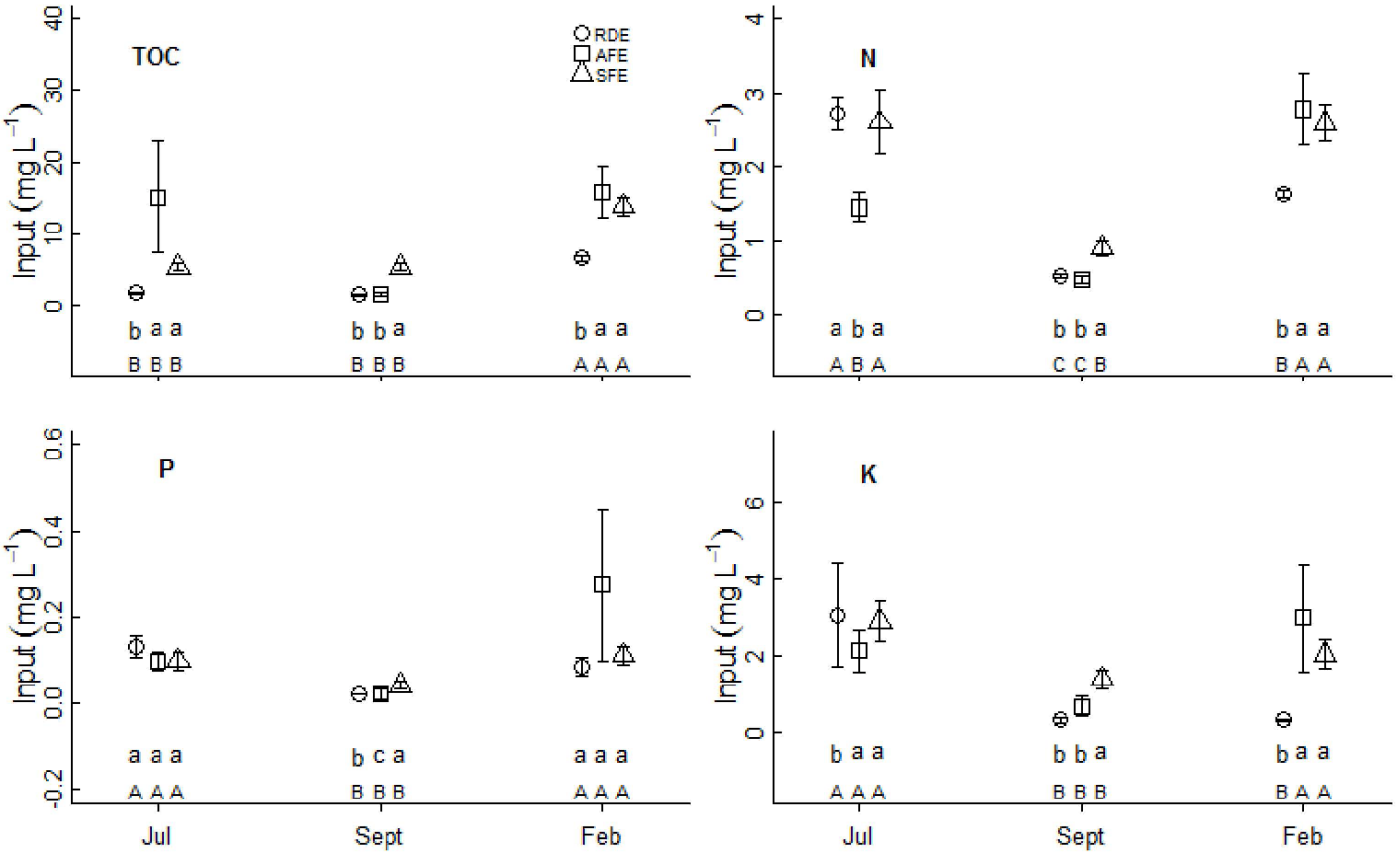
Organic carbon and nutrient concentrations (mean ± standard error) in input water. RDE, AFE, SFE are as defined in the text. Different lowercase letters indicate significant differences between two systems (*P*<0.05); different uppercase letters indicate significant differences between two seasons (*P*<0.05) in the same ecosystem.

**Fig 4:**
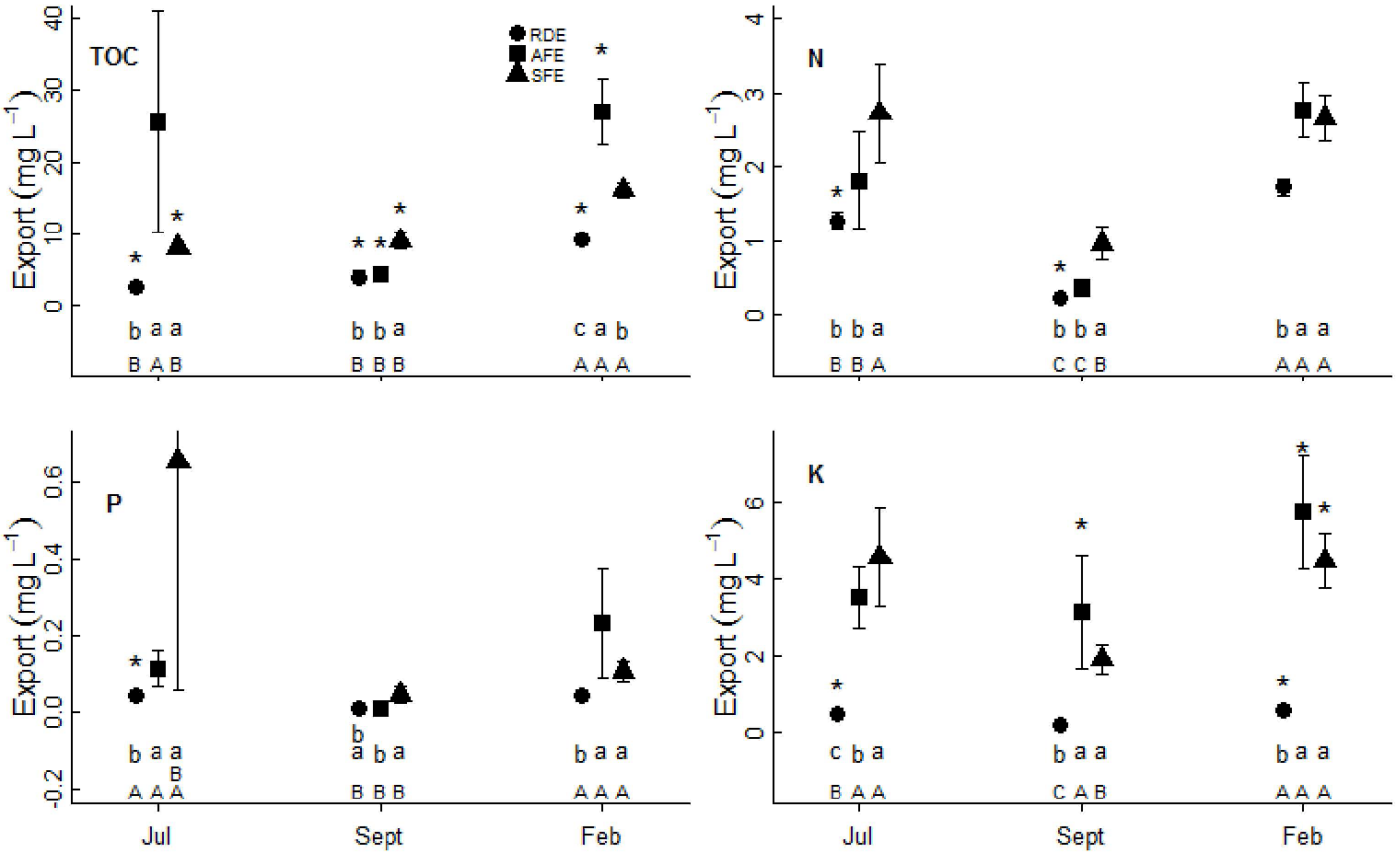
Organic carbon and nutrient concentrations (mean ± standard error) in exported water. All designations are as above, except * represents a significant difference (*P*<0.05) between input (Fig 3) and export.

TOC, N, P, and K were determined in both the water received by rock outcrops and in water exported to nearby soil patches at all three sampling times in the three ecosystems (Figs 3 and 4). On average, 7.47±0.77 mg L^-1^ organic carbon, 1.74±0.09 mg L^-1^ N, 0.10±0.01 mg L^-1^ P, and 1.74±0.18 mg L^-1^ K were found in water received by rock outcrops, and 11.82±1.38 mg L^-1^ organic carbon, 1.62±0.11 mg L^-1^ N, 0.14±0.05 mg L^-1^ P, and 2.73±0.25 mg L^-1^ K were measured in water exported to nearby soil patches. Both the water received by rock outcrops and exported to soil patches varied across the three sampling times representing different seasons, and across ecosystems. Generally, organic carbon and nutrient concentrations were highest during the dry season (February), and were higher in the forested ecosystems (AFE and SFE) than in the rock desertification system (RDE: Figs 3 and 4). The organic carbon and nutrient concentrations of exported water changed consistent with the carbon and nutrient concentrations of the inputs. Paired t-tests showed that TOC and K concentrations were significantly higher in water exported than in input samples at most sampling times across the three systems, while N and P were lower or remained unchanged (Figs 3 and 4).

TOC and nutrient concentrations of exports were correlated positively with those of inputs for at least one of the three sampling times. In addition, the concentration of TOC was correlated negatively with the quantity of water output per unit of rock projected area. Further, the concentration of N was correlated positively with the sampled surface area to catchment area ratio, and the concentration of K was correlated positively with the quantity of organic carbon in epiliths on 30^th^ June. However, TOC and nutrient concentrations in exports did not correlate with rock slopes, nor with the quantity of water input per unit of rock projected area (Table 3).

**Table 3:** Factors correlated with organic carbon and nutrient concentrations in outcrop runoff water and their significance levels at different times.

		Estimate			
Factors	Time	TOC	N	P	K
Corresponding organic carbon and nutrient concentrations in input water	30^th^ Jun	0.46*	0.41*		
Corresponding organic carbon and nutrient concentrations in input water	1^st^ Sep	0.54**	0.63***		0.64***
Corresponding organic carbon and nutrient concentrations in input water	18^th^ Feb	0.50**		0.39*	0.72***
Water output quantity per unit of rock projected area	30^th^ Jun	-0.44*			
Ratio of sampled surface area to catchment area	30^th^ Jun		0.40*		
Ratio of sampled surface area to catchment area	18^th^ Feb	NA			NA
Quantity of organic matter in epiliths per m^2^ of surface area	30^th^ Jun				0.48**

The analyses (PCAs) were conducted testing the influence in each season. NA means that the data failed to meet the prerequisites for analyses.

* means *P*<0.05

** means P<0.01

*** means *P*<0.001

### Annual input and output fluxes of organic carbon and nutrients on the rock surface

Both input and export of annual organic carbon and nutrients per m^2^ of rock projected area varied among the three ecosystems; however, differences between any two were seldom significant (Table 4). Only the input and export of TOC and the export of K in the RDE were significantly lower than that in the AFE and the SFE. Over the year, on average, 5.18±0.96 g m^-2^ organic carbon, 1.69±0.12 g m^-2^ N, 0.08±0.01 g m^-2^ P, and 1.80±0.34 g m^-2^ K were received from water input by rock outcrops, and 3.39±0.36 g m^-2^ organic carbon, 0.64±0.13 g m^-2^ N, 0.12±0.09 g m^-2^ P, and 1.01±0.27 g m^-2^ K were exported to nearby soil patches via runoff (Table 4). Paired t-test results demonstrated that N and P exports were significantly smaller than were N and P inputs in all three ecosystems, and that the export of TOC in the SFE and that of K in the RDE and AFE were significantly smaller than were those of the input (Table 4).

**Table 4:** Annual TOC and nutrients received by rock outcrops and exported to nearby soil patches per m^2^ of rock projected areas (mean ± standard error) in the three karst ecosystems.

Input/ Export	Ecosystems	TOC (g m^-2^)	N (g m^-2^)	P (g m^-2^)	K (g m^-2^)
Input	RDE	2.10±0.11 b	1.63±0.10 a	0.07±0.008 a	1.75±0.82 a
Input	AFE	8.29±2.87 a	1.46±0.25 a	0.10±0.05 a	1.64±0.54 a
Input	SFE	5.87±0.47 a	1.97±0.23 a	0.08±0.01 a	2.00±0.29 a
Export	RDE	1.99±0.20 b	0.44±0.03 a*	0.01±0.001 a*	0.15±0.03 b*
Export	AFE	4.32±0.78 a	0.46±0.10 a*	0.04±0.02 a*	1.03±0.25 a*
Export	SFE	4.11±0.58 a*	1.04±0.34 a*	0.30±0.27 a*	1.96±0.65 a

RDE: rock desertification ecosystem; AFE: anthropogenic forest ecosystem; SFE: secondary forest ecosystem. Different lower case letters indicate significant differences between two systems (*P*<0.05)

* represents a significant difference (*P*<0.05) between input and export in the same ecosystem

In the RDE, the annual inputs of TOC, N, P, and K to outcrops per m^2^ of rock projected area accounted for very high ratios of these nutrients in epilithic pools, while in forests (AFE and SFE), these ratios decreased (Table 5). The annual TOC, N, and P exports of outcrops to those of the epilithic pool were ordered as follows: RDE > AFE > SFE, while the annual K export to the epilithic pool proceeded in the following order: RDE > SFE > AFE (Table 5).

**Table 5:** Ratios of the annual input of total organic carbon (TOC) and nutrients per m^2^ of rock projected area (*INP*) to those of the epiliths pool (*POOL*), ratios of annual runoff exportation of TOC, and nutrients per m^2^ of rock projected area (*EXP*) to those of the annual input (*INP*) for the three ecosystems.

Ratios	Ecosystems	Organic carbon	N	P	K
(*INP*)/(*POOL*)	RDE	0.22:1	1.65:1	1.09:1	12.5:1
(*INP*)/(*POOL*)	AFE	0.09:1	0.21:1	0.24:1	0.56:1
(*INP*)/(*POOL*)	SFE	0.02:1	0.08:1	0.03:1	0.65:1
(*EXP*)/(*INP*)	RDE	0.95:1	0.27:1	0.14:1	0.09:1
(*EXP*)/(*INP*)	AFE	0.52:1	0.32:1	0.40:1	0.63:1
(*EXP*)/(*INP*)	SFE	0.70:1	0.53:1	3.75:1	0.98:1

RDE: rock desertification ecosystem; AFE: anthropogenic forest ecosystem; SFE: secondary forest ecosystem.

Based on the annual input and export flux of organic carbon and nutrients, the contribution of TOC, N, P, and K to nearby soil patches can be estimated at different rock outcrop emergence ratios. At a 30% emergence ratio, TOC, N, P, and K received by soil patches in a certain area from rock runoff in the three ecosystems amounted to 4%-161% of those received from direct atmospheric deposition. When the emergence ratio approached 70%, the percentage was 21%-875% of the amount received from precipitation (Table 6). The percentages assumed the following order: SFE > AFE > RDE for N, P, and K; for TOC, however, the order was RDE > SFE > AFE.

**Table 6:** Estimated ratios of TOC and nutrients received annually from rock runoff water to those received from atmospheric deposition by soil patches with different outcrop to soil area ratios (R/S) in the three ecosystems.

		In rock runoff water/atmospheric deposition			
R/S	Ecosystems	TOC	N	P	K
3:7	RDE	0.41:1	0.12:1	0.06:1	0.04:1
3:7	AFE	0.22:1	0.14:1	0.17:1	0.27:1
3:7	SFE	0.30:1	0.23:1	1.61:1	0.42:1
7:3	RDE	2.22:1	0.63:1	0.33:1	0.21:1
7:3	AFE	1.21:1	0.75:1	0.93:1	1.47:1
7:3	SFE	1.63:1	1.24:1	8.75:1	2.29:1

RDE: rock desertification ecosystem; AFE: anthropogenic forest ecosystem, SFE: secondary forest ecosystem; R/S: presumed rock area to soil area ratios.

## Discussion

### Organic carbon and nutrients received by and exported from rock surfaces

Karst rock outcrops collected organic carbon and nutrients, after which a large proportion of these were exported to nearby soil patches (Figs 3 and 4, Table 3). To our knowledge, to date, no field data have quantified exports from rock outcrops in karst landscapes directly. In a simulated experiment on a rocky, semi-arid, Mediterranean hill slope, Lange et al. [28] found that not all water from outcrops reached nearby soil patches, and instead, that it was likely that during heavy rainfall, most of the water rushed down the rock surface and drained away in the form of land surface runoff. Even though, other studies have implied that rock outcrops’ exports in various rock-outcrop landscapes do affect the nearby soil patches. For example, rocks in a pristine glacial forefield site were found to funnel N and P to nearby soils and to lead to heterogeneous patterns of soil fertility [9]. Further, the N content in the topsoil increased with proximity to the base of inselbergs in savannas in both South Africa and Venezuela [8]. In some desert ecosystems, hypolithic colonization of microbial communities beneath pebbles can extend into surrounding soils and increase soil biomass and fertility [6], and in the Negev desert, cobbles may serve as a nutrient sink by condensing dew [7]. Liu [29] found that the amount of total N and organic carbon in certain soil patches in severe rock desertification sites (where rock outcrops constitute more than 70% of the total area) differed from, or were even higher than, in those of less severely degraded sites in Guizhou, a neighboring province with a similar karst landscape, and inferred the export contribution of rock outcrops. Similar results were obtained in Guangxi (a neighboring province with a similar karst landscape) by Zhang et al. [30], who indicated that higher proportions of rock outcrops were related positively to soil organic carbon, total N and P, available N and K, and C/N ratios, which the authors attributed in part to the role that epiliths, such as bryophytes and cyanobacteria, play in improving soil fertility; this finding has been supported by a number of other studies [31,32].

### Factors correlated with runoff organic carbon and nutrients

Both the concentrations and the annual total amounts of organic carbon and N, P, and K in exported water were linked with those of water input to rock outcrops. PCAs showed that the concentrations of TOC, N, P, and K in runoff water were correlated positively with those in water received by the rock outcrops at a given collection time (Table 3), and in most cases, annual inputs changed in tandem with exports (Table 4). However, a direct comparison showed that TOC and K were enriched in exported water compared with input water, while N and P were depleted or remained unchanged (Figs 3 and 4). These findings indicated that rock outcrops play different roles when TOC, N, P, and K pass through. These responses are parallel in part to a number of rain chemistry studies in canopies [33,34], which showed that structural elements such as N are less likely to be leached relative to those seen commonly in the cell solution, such as K [35]. Thus, we expected the possible variation in nutrient concentration in input water and subsequently in runoff water at other karst sites.

The amount of organic carbon and nutrients received by rocks and exported to nearby soil patches was dynamic, both seasonally and across different ecosystems (Figs 3 and 4, Table 4). These variations may be related to inputs, leaching and holding capacity, contributions of the rock surface, and the interplay of biotic and hydrological processes [36]. Atmospheric deposition may play an important role in controlling nutrients received by rock outcrops, and canopy leaching also provides carbon and nutrients [33,35,37]. Forested ecosystems (AFE and SFE) had higher concentrations of organic carbon and nutrients in the input and runoff water when compared with the RDE (Figs 3 and 4). The abundant epiliths and detritus of all types on the rock surface are assumed to enrich the runoff, just as the canopy enriches throughfall [38], and the slope of the rock surface and the amount of water runoff from the rock surface may influence export concentration as well. However, PCAs did not find a strong correlation between export concentrations with epiliths, rock slope, and water runoff from the rock surface. This finding was attributed to the steep slope of the rock and the limited variation in slope among the three ecosystems, which did not differ significantly. The steep slope of ≥ 66° (Table 1) may conceal other effects of epiliths. Thus, neither the effect of the slope nor the quantity of water runoff, which may be influenced strongly by the slope, were significant.

The net retention of ions from the canopy typically is calculated by subtracting the fluxes in throughfall and stem flow (output) from the total deposition (input) [34]. Following this pattern, we regard rock outcrops as sub-ecosystems in karst landscapes, and treating atmospheric deposition (*INP*) as an input of TOC, N, P, and K; exports (*EXP*) as an output; and epilithic amounts as parts of the pool (*POOL*). However, net retention of organic carbon and nutrients by rock surfaces between the *EXP* and *INP* was not found to be equal to the amounts held by epiliths, as some of the organic carbon and nutrients may have been lost to cracks in the rocks and via splashes, which are difficult to quantify. The collection system in this study also had its limitations in ensuring a closed balance between input and output of the organic carbon and nutrients on the rock surface. This finding indicates that the organic carbon and nutrients in the input and export water are an important, albeit limited, component of the knowledge required to trace the exchange processes of organic carbon and nutrients between flowing water and rock surfaces. However, as losses through cracks and splashes in the same area are unlikely to vary greatly, the ratio of *INP*/*POOL*, and *EXP*/*INP* can serve as indicators of the role of rock outcrops in capturing TOC, N, P, and K. The annual ratio of organic carbon and nutrient input to the epilithic pool per m^2^ of projected area (*INP*/*POOL*) took the following order: RDE > AFE > SFE. In contrast, the ratio of N, P, and K exported by the annual runoff by rock per m^2^ of projected area to input (*EXP*/*INP*) assumed the following the order: SFE > AFE > RDE (Table 5). This order implies that a higher ratio of atmospheric deposition was fixed by rock outcrops in the RDE, and a higher ratio of the atmospheric deposition of TOC, N, P, and K was exported to nearby soil patches in the forests (SFE, AFE).

### Ecological significance of water runoff from outcrops

**Fig 5:**
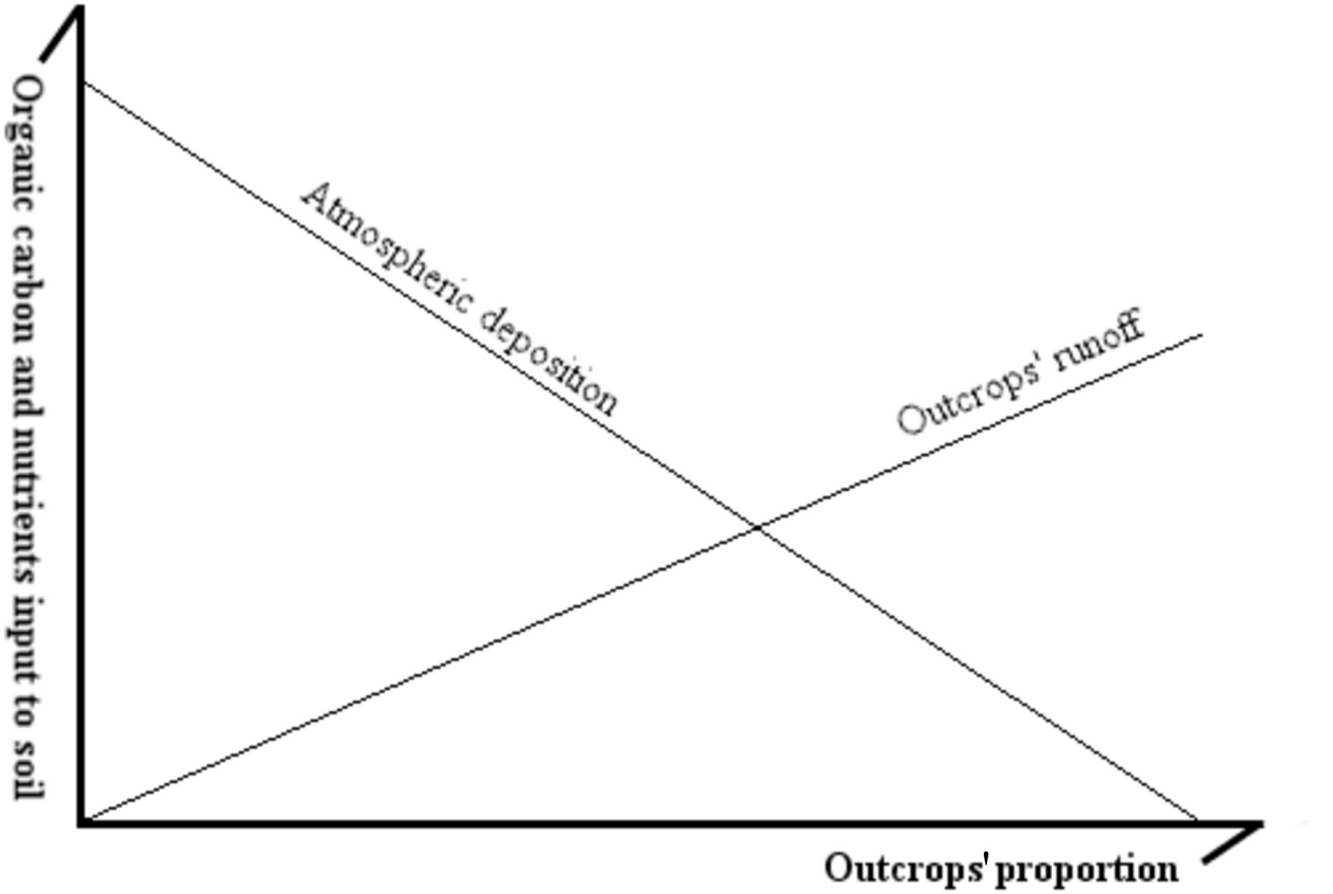
Theoretical model showing the amounts of organic matter and nutrients received by soil patches from atmospheric deposition and from rock outcrops’ runoff along an outcrop’s proportion gradient.

Soil patches received TOC, N, P, and K from direct deposition and runoff exported from surrounding rock outcrops. In areas in which the proportion of rock is low, the runoff amount also is low and can be ignored, but when the rock-to-soil patch area ratio reaches 3:7 (the lower criterion of rock desertification presented by the National Forest Bureau of China, Technology Regulations of Vegetation Restoration in Karst Desertification Zone, LY/T 1840–2009) or higher, the TOC, N, P, and K released from the outcrops may account for a considerable proportion of the total carbon and nutrients that soil patches receive by comparison with those received from direct atmospheric deposition. Under most circumstances, when the rock-to-soil patch area ratio is 7:3 or higher, the contribution of rock outcrops likely exceeds the contribution of deposition (Table 6, Fig 5). Given this situation, annual runoff exports of TOC, N, P, and K to the soil by rock outcrops may approach 1392±137–3026±546 kg km^−2^ (export flux in Table 4 × 10^6^ m^2^ × 70%), 308±24–725±237 kg km^−2^, 10±1–208±190 kg km^−2^, and 108±18–1369±521 kg km^−2^, respectively, among the three ecosystems. This amount will result in a greater increase in TOC, N, P, and K input to the other 30% of soil patches.

From these results, we concluded that the emergence of rock outcrops does not necessarily result in lower fertility within nearby soil patches. If rock outcrops result from soil erosion, this phenomenon will give rise to low productivity within the ecosystem overall. However, runoff inputs of water and fertilizers are sufficient to sustain a large tree, which is referred to as the “flowerpot effect” in the karst region. Because karst land consists of “flowerpots” of various sizes, the distribution of organic carbon, nutrients, and water from rock outcrops to nearby soil patches may also increase heterogeneity in soil fertility. Across sites and seasons, the amount of rock runoff into nearby soil patches will vary considerably. This variation arises because other hydrologic components (crack and fissure loss, splashing from the rock surface, and rock surface interception) combine with rock runoff to divert the organic carbon and nutrients contained in water inputs. Moreover, conditions are influenced by changing rain characteristics [28] and the unique traits of the karst environment, such as various rock outcrop morphologies, sizes, and surface conditions, variations in vegetation cover, the diversity in richness and extent of organisms that inhabit rocks, and different ratios of rock to soil area. Habitat heterogeneity is assumed to contribute to biodiversity in karst regions [39,40], such that more plant species are likely to become established as a result of spatially diversified soil conditions. Clements et al. [39] suggested that the rugged terrain and edaphic isolation formed by karst rock outcrops function as an “ark of biodiversity.”

Data in this study likely underestimated the proportion of TOC, N, P, and K exported to soils from surrounding rock outcrops via runoff, because we were unable to measure splash loss from the rock during heavy rainfall. Moreover, the free fall of debris from dead organisms may occur at any time; therefore, these were also beyond our ability to detect in the thrice-yearly collections. Runoff exports of critical resources by rock outcrops also may be highly variable across different geological locations, ecosystems, precipitation events, and the like. We suggest that further research is needed to target different ecosystems and different rock-soil distribution patterns. Taking additional biotic and abiotic factors into consideration, and sampling more frequently would enhance our understanding of these complex systems.

Karst landscapes constitute approximately 12–15% of the global terrestrial surface [1–3]. This study revealed that outcrops function as an important medium to receive and store atmospheric organic carbon and nutrients, and redistribute large proportions of them into soil via rock runoff. Runoff-borne organic carbon and nutrients might be affected by some biotic and abiotic factors, such as epilithic biomass, rock fissures, quantity of output water, and rock slope, among others. The redistribution of organic carbon and nutrients to soil patches under different proportions of outcrops would increase the heterogeneity of soil fertility, and exert a profound effect on karst biodiversity.

## Supporting Information

S1 Table Concentration of TOC and nutrients for water samples collected in three seasons. (XLSX)

S2 Table Surface area and projected area of sampling rocks. (XLSX)
